# Patterns of acute inflammatory symptoms prior to cancer diagnosis

**DOI:** 10.1038/s41598-017-00133-8

**Published:** 2017-03-06

**Authors:** Andrea Setiawan, Li Yin, Gert Auer, Kamila Czene, Karin E. Smedby, Yudi Pawitan

**Affiliations:** 1 School of Pharmacy, University of California, San Francisco, USA; 2 Department of Medical Epidemiology and Biostatistics, Karolinska Insitutet, Stockholm, Sweden; 3 Department of Oncology and Pathology, Karolinska Insitutet, Stockholm, Sweden; 4 Unit of Clinical Epidemiology, Department of Medicine Solna, Karolinska Insitutet, Stockholm, Sweden

## Abstract

Although many studies have examined the role of chronic inflammation in cancer development, few studies discuss the patterns of acute inflammation prior to cancer diagnosis. Patients with lung, colorectal, prostate, or breast cancer between 1 July 2006 and 31 December 2009 and their metastatic status at diagnosis were determined through the Swedish Cancer Register. Non-steroidal anti-inflammatory drugs (NSAIDs) use in the year prior to cancer diagnosis was assessed through the Swedish Prescribed Drug Register. There were 13,945 patients identified with breast cancer, 6501 with prostate cancer, 5508 with lung cancer, and 12,723 with colon cancer. For metastatic patients, there is strong evidence of higher NSAIDs use 1–3 months compared to 10–12 months prior to diagnosis (breast odds ratio (OR) = 3.54, 95% CI 2.26–5.54; prostate OR = 3.90, 95% CI 3.10–4.90; lung OR = 2.90 95% CI 2.44–3.44; colorectal OR = 1.67, 95% CI 1.36–2.05). For non-metastatic patients, increased NSAIDs use 1–3 months prior to diagnosis was also observed, but only to a smaller extent for lung and prostate cancer (prostate OR = 1.48, 95% CI 1.27–1.72; lung 1.41, 95% CI 1.19–1.67). In conclusion, if NSAIDs use reflects underlying inflammatory symptoms, there is support for the hypothesis that advanced cancer was associated with an acute inflammatory process.

## Introduction

The link between chronic inflammation and tumor progression has been studied extensively. Many studies have examined the effects of chronic inflammation in initiating tumorigenesis through pro-inflammatory mediators, which promote cell proliferation and angiogenesis^1–3^. On the other hand, there is increasing evidence that neoplastic cells promote themselves through constitutive secretion of pro-inflammatory cytokines, which prolong inflammation at the primary site and create a tumorigenic microenvironment^4, 5^. Genetic alterations in oncogenes, such as RAS and MYC, might produce neoplastic cells that constitutively secrete cytokines, such as IL-6 and TNFα, contributing to an inflammatory microenvironment^4^. Several studies have also indicated that systemic inflammation in cancer patients was associated with worse survival^6, 7^, thus motivating the clinical use of inflammatory markers as prognostic indicators. Biologically it is likely that the two directions in the link between inflammation and cancer are not mutually exclusive with the exact nature depending on the type of cancer^5^.

Despite the extensive studies discussing the relationship between chronic inflammation and cancer, few studies discuss symptoms *prior* to diagnosis that develop due to these inflammatory processes. Currently, the majority of studies examine symptoms of patients with an advanced stage of cancer *post* diagnosis, particularly symptoms during treatment. Ott *et al*.^8^ notes that most cancers are initially asymptomatic, so without an early screening program they may get diagnosed at an advanced stage, such as in lung cancer patients, however, we aim to investigate the hypothesis that acute and nonspecific inflammatory symptoms emerge when cancer is at advance stage. If true, then we should see more use of anti-inflammatory drugs prior to diagnosis of advanced cancers.

Non-steroidal anti-inflammatory drugs (NSAIDs) have become one of the most commonly prescribed drug groups due to their anti-inflammatory properties and have been prescribed to alleviate pain symptoms in cancer patients^9, 10^. The therapeutic properties of NSAIDs for cancer patients have gained interest as pain and swelling are common early inflammatory symptoms associated with some cancers^8, 11^. Here we study the patterns of anti-inflammatory use in the year prior to cancer diagnosis to reveal when and to what extent inflammatory symptoms emerge in patients who will be diagnosed with an advanced stage of cancer.

## Results

The number of individuals in the control group and in each cancer, and their main characteristics are given in Table 1. The median age at diagnosis of cancer is range from 68 to 71 years, with the exception of breast cancer patients at 62 years. Lung cancer patients show the lowest proportion having high education, which could be due to higher rate of smoking among the lower-educated population.

**Table 1:** Characteristics of the study population.

	Control	Breast	Prostate	Colorectal	Lung
	N = 198,240	13,945	6,501	12,723	5,508
Age (%)					
<55	16.5	30.4	3.6	10.1	9.1
55–69	30.2	41.0	46.5	34.7	49.2
70–84	44.2	23.0	44.9	45.7	39.6
= > 85	9.2	5.7	5.0	9.4	2.1
Median (years)	71	62	70	71	68
Male (%)	51.7	0	100	52.4	49.8
Educational level (%)					
Low	33.3	20.1	36.4	36.7	36.5
Mid	45.2	49.3	43.2	44.1	49.6
High	21.6	30.6	20.4	19.2	13.9

For all four cancers, metastatic patients showed an increased use of NSAIDs 1–3 months compared to 10–12 months prior to diagnosis (Table 2) (breast odds ratio (OR) = 3.54, 95% confidence interval (CI) 2.26–5.54; prostate OR = 3.90, 95% CI 3.10–4.90; lung OR = 2.90, 95% CI 2.44–3.44). In the case of metastatic colorectal cancer patients, the increased use of NSAIDs 1–3 months prior to diagnosis was less pronounced (OR = 1.67, 95% CI 1.36–2.05). For non-metastatic disease, only the prostate and lung cancer patients had an increase NSAIDs use in the 1–3 months prior to diagnosis (prostate OR = 1.48, 95% CI 1.27–1.72; lung OR = 1.41, 95% CI 1.19–1.67). For these two cancers we also observed a smaller increase in drug use 4–6 months prior to diagnosis of metastatic disease (prostate OR = 1.48, 95% CI 1.15–1.92; lung OR = 1.28 95% CI 1.06–1.55). None of these increases was observed in the control group.

**Table 2:** Patterns NSAIDs use in the year prior to cancer diagnosis.

Breast				Prostate			
**Total**	**Controls**	**M0**	**M1**	**Total**	**Controls**	**M0**	**M1**
	95,779	13551	394		102,461	4726	1775
**1–3 mo**	6906	1082	86	**1–3 mo**	6029	450	351
	0.97(0.94, 1.00)	1.03(0.94, 1.12)	3.54(2.26, 5.54)		0.96(0.92, 0.99)	1.48(1.27, 1.72)	3.90(3.10, 4.90)
**4–6 mo**	7019	1100	35	**4–6 mo**	6023	360	154
	0.98(0.95, 1.02)	1.05(0.96, 1.14)	1.23(0.74, 2.06)		0.96(0.92, 0.99)	1.16(0.99, 1.36)	1.48(1.15, 1.92)
**7–9 mo**	7184	1057	33	**7–9 mo**	6009	315	122
	1.01(0.98, 1.05)	0.92(0.90, 1.09)	1.15(0.68, 1.95)		0.95(0.92, 0.99)	1.00(0.85, 1.18)	1.15(0.88, 1.51)
**10–12 mo**	7117	1056	29	**10–12 mo**	6284	314	107
	1 (reference)	1 (reference)	1 (reference)		1 (reference)	1 (reference)	1 (reference)
**Lung**				**Colorectal**			
**Total**	**Controls**	**M0**	**M1**	**Total**	**Controls**	**M0**	**M1**
	198,240	2952	2556		198,240	9759	2964
**1–3 mo**	12935	355	530	**1–3 mo**	12935	499	256
	0.96(0.94, 0.99)	1.41(1.19, 1.67)	2.90(2.44, 3.44)		0.96(0.94, 0.99)	1.01(0.89, 1.14)	1.67(1.36, 2.05)
**4–6 mo**	13042	281	261	**4–6 mo**	13042	548	173
	0.97(0.95, 1.00)	1.08(0.911, 1.29)	1.28(1.06, 1.55)		0.97(0.95, 1.00)	1.11(0.98, 1.26)	1.09(0.88, 1.37)
**7–9 mo**	13193	258	195	**7–9 mo**	13193	553	169
	0.98(0.96, 1.01)	0.98(0.82, 1.18)	0.90(0.73, 1.11)		0.98(0.96, 1.01)	1.12(0.99, 1.27)	1.07(0.85, 1.33)
**10–12 mo**	13401	262	214	**10–12 mo**	13401	496	159
	1 (reference)	1 (reference)	1 (reference)		1	1 (reference)	1 (reference)

The entries for each period in each table show the number of drug users in the period, the odds ratio (OR) and 95% confidence interval. The OR compares the drug use during each period with the 10–12 month period as reference. The analysis is adjusted for age, sex and education level.

Lymph-node status was adjusted for in the next analyses in order to assess whether the NSAIDs use could also be explained by the cancer having spread to the lymphatic nodes. There was no such evidence, as the lymph-node adjusted results (Supplementary Table 1) are all very close to the previous results in Table 2. For example, after adjusting for node status, for non-metastatic prostate and lung cancer patients, there was an increased drug use within the 1–3 months prior to diagnosis (prostate OR = 1.48, 95% CI 1.27–1.72; lung OR = 1.41, 95% CI 1.19–1.67).

For colorectal cancer, we performed additional analyses to adjust for known inflammatory conditions (Method Section) and, as a sensitivity analysis, to extend the exposure period to two years prior to diagnosis. The results in Supplementary Table 2 show that all the previous results hold, with the increased use of NSAIDs being observed only 1–3 months prior to metastatic diagnosis.

To test whether the pattern of increased drug use in the year prior to cancer diagnosis unique to NSAIDs, the same analysis was done with metformin and statin. None of these drugs showed any increase use near the time of diagnosis of any cancer (Supplementary Tables 3 and 4).

Finally, to assess the direction of association between the use of anti-inflammatory and metastasis, we followed those diagnosed with non-metastatic disease until death or the last follow-up date. Exposure was defined as the anti-inflammatory use within 6 months prior to diagnosis, and the endpoint was overall survival. The numbers of non-metastatic patients in different cancers and the numbers exposed can be seen in Table 2. The total numbers of deaths during follow-up were 942 for breast cancer, 402 for prostate cancer, 1630 for lung cancer and 1921 for colon rectum cancer. For all cancers, we did not observe any association between the exposure and overall survival after cancer diagnoses: breast hazard ratio (HR) 0.87, 95% CI 0.71–1.07; prostate HR = 0.88, 95% CI 0.66–1.18; lung HR = 1.01, 95% CI 0.88–1.15; colorectal HR = 1.04, 95% CI 0.89–1.23. The survival curves are shown in Supplementary Figure 1S.

## Discussion

Taken together, our results indicate that (i) metastatic breast, prostate and lung cancer patients had an acute 3- to 4-fold increase in NSAIDs use 1–3 months compared to 10–12 months prior to diagnosis. Colorectal cancer patients showed lower increase. (ii) For non-metastatic patients, there was also an increase of drug use, but only to a smaller extent for lung and prostate cancer patients. (iii) None of these increases was observed in the control group or with the use of statin and metformin, indicating that the phenomenon was specific to the use NSAIDs prior to cancer diagnosis. (iv) For non-metastatic patients, we also did not observe any association between drug use within 6 months prior to diagnosis and overall survival after diagnosis.

The interpretation of these results depends on two key issues: (i) direction of causality and (ii) the reasons for NSAID use. For the former, either the increase drug use is due to the underlying undiagnosed metastatic disease (i.e. reverse causality), or the increase drug use causes cancer metastasis (forward causality). If the latter was true then, among the non-metastatic patients, we should see an association between drug use prior to diagnosis and worse survival after diagnosis. As we did not see this association, the reverse causality appears to be a more likely explanation. In analyses of drug use, this reverse causality is well known as confounding by indication.

Next, how can we interpret the NSAIDs use itself? They must have been used to relieve nonspecific symptoms, which could be (i) pain or discomfort due to the simple presence of an undiagnosed cancer in the relevant organ, or (ii) an acute inflammation, which, by the preceding argument, is promoted by the cancer. Our data cannot completely rule out the first reason. In their detailed analyses, García Rodríguez and González-Pérez^12^ did not observe any increase in current use of the analgesic paracetamol at the time of breast cancer diagnosis; they in fact found the opposite, i.e. fewer current users among the cancer cases. This result indicates that pain alone could not explain the increase use of NSAIDs prior to cancer. We also note that the increase in drug use was primarily and to a larger extent observed in metastatic patients rather than in non-metastatic patients, implying that nonspecific symptoms are more likely to develop at advanced stages of cancer rather than at earlier stages. Biologically, the inflammatory milieu, which consists of neoplastic cells and immune cells, plays an important role in facilitating metastasis by initiating the epithelial-mesenchymal transition (EMT) process^3^. During the EMT process, immune cells within the tumor microenvironment secrete inflammatory cytokines, growth factors, and proteases, which attenuate cell-cell adhesion by reducing E-cadherin expression and make neoplastic cells mobile^2, 3^.

Because NSAIDs can inhibit cyclooxygenase enzyme 1 (COX-1), which produces prostaglandins that maintain the gastrointestinal mucosal lining^10^, NSAIDs use is normally contraindicated in patients with a history of ulcers or IBD. However, even after adjusting for this and extending the exposure period to two years, there was still an increase use of NSAIDs in the 1–3 months prior to diagnosis of metastatic colorectal cancer. Because we are comparing the drug use across time periods within, say, metastatic patient group, we note that there is no conflict between our results with previous studies that showed the association between the NSAIDs use and a reduced risk of colorectal cancer^13–15^. The latter analysis would involve using the control group as the reference group.

The main strength of our study is that it is a large unbiased population-based study, thus capturing the experience of a large unselected group of individuals, over four most common cancers. One weakness of this study is the inability to account for over-the-counter NSAIDs use prior to cancer diagnosis. If such use was included, one likely outcome was that the effect size would be more pronounced and detected earlier than 3 months for most cancers. In addition, because this is an observational study, there may be unmeasured confounders. However, because the exposure (periods prior to cancer diagnosis) is externally defined for all individuals, i.e. it is not subject to an individual’s decision, so it is not likely to be causally related or confounded to other factors. For example, this exposure cannot be associated with life-style choices.

In summary, we have observed an acute increase of NSAIDs use prior to diagnosis of primarily metastatic cancer. If NSAIDs use reflects underlying inflammatory symptoms, there is support for the hypothesis that advanced cancer may promote an acute inflammatory process.

## Methods

### Cancer Incidence

The Swedish Cancer Register was created in 1958 and contains individuals with histologically confirmed cancer. The register covers 95% of all cancer cases and in 2004 began including TNM status which refers to the tumor extent (T), nodal involvement (N) and metastatic status (M) at diagnosis, however, our study focused on M status. Primary cancer patients with a biopsy based diagnosis between 1 July 2006 and 31 December 2009 were included in the study while patients that had previous cancer were excluded. During this time period, there were 13,945 breast, 6,501 prostate, 5,508 lung and 12,723 colorectal cancer identified.

Our study was part of a population-register based study approved by the Stockholm Regional Ethical Committee.

### Control Group

Negative controls were selected from the Total Population Register for each cancer patient identified during the study period (including all cancer types, not just breast, prostate, lung and colorectal cancer). These controls were cancer-free during the calendar year at diagnosis of the corresponding cancer case; they were matched on gender and age^16^. A total of 198,240 control subjects was selected, including 95,779 women and 102,461 men.

### NSAIDs prescription and timing

The National Prescribed Pharmaceutical Register was used to determine the use of prescribed NSAIDs. Users were defined as those who used NSAIDs with Anatomical Therapeutic Chemical (ATC) codes M01AB or M01AE; these two classes cover approximately 90% of all NSAIDs use. Separate analyses of these classes of drugs did not reveal any distinct pattern, so all of following results refer to the combined M01AB and M01AE users. The year prior to cancer diagnosis was subdivided into 3 month intervals, resulting in the exposure categories of 1–3 months, 4–6 months, 7–9 months, and 10–12 months. In all analyses the last period is used as the reference category. As a sensitivity analysis (given in the Supplementary report), we performed an analysis of colorectal cancer using the period 22–24 months prior to diagnosis as the reference category.

As negative controls for the NSAIDs, we also performed similar analyses for statin and metformin; the former is indicated for hypercholesterolemia, while the latter is for diabetes, so we should not observe any increase in usage prior to cancer diagnosis. The results are given in the Supplementary report.

### Adjustment for potential confounders

There were a number of factors that could have confounded the use of NSAIDs prior to cancer. Age (from the Cancer Register was divided into 5 year intervals), sex (taken from the Cancer Register) and education (obtained from the Education Register and categorized as low, middle, or high) were included in the analyses to adjust for any possible confounding. In addition, we also adjusted for node status to determine whether the inflammatory symptoms were due to the cancer spreading to lymphatic nodes rather than the distant metastases (Supplementary report).

NSAIDs use would be contraindicated in patients with a history of ulcers or IBD, so for colorectal patients we performed additional analyses (Supplementary report), where we adjusted for gastrointestinal ulcers and inflammatory bowel disease (IBD), including Crohn’s disease and ulcerative colitis based on international disease classification (ICD) codes K25–29 and K50-K51 obtained from the hospitalization Register.

### Statistical Analysis

The primary outcome for each individual was a vector of repeated observations of drug use in each 3-month period prior to diagnosis of cancer. To allow for correlations between the outcomes within the same individual, we used the generalized estimating equation with robust variance formula, using the drug use as outcome and period as exposure. Logit link was used to in order to calculate odds ratios (ORs) of drug use between each period to the 10–12 month period as reference. We adjusted the analyses for possible confounders as described above. Adjusted ORs and their 95% confidence intervals (CIs) are reported for the controls, the non-metastatic and the metastatic patients separately.

## Electronic supplementary material

Supplementary Tables
