# Supplementary material for: Patterns of acute inflammatory symptoms prior to cancer diagnosis
Source: Sci Rep. 2017 Mar 6;7:67. doi: 10.1038/s41598-017-00133-8 (PMC5427907; doi:10.1038/s41598-017-00133-8)

Supplementary Report

Patterns of acute inflammatory symptoms prior to cancer diagnosis

Andrea Setiawan, Li Yin, Gert Auer, Kamila Czene, Karin E. Smedby, Yudi Pawitan

Correspondence to: Yudi Pawitan, Department of Medical Epidemiology and Biostatistics, Nobels väg 12A, Karolinska Institutet, 17177 Stockholm, Sweden. Email: yudi.pawitan@ki.se

Affiliation of authors: Department of Medical Epidemiology and Biostatistics (YP, LY, KC), Unit of Clinical Epidemiology, Department of Medicine Solna (KES), and Department of Oncology and Pathology (GA), Karolinska Institutet, Stockholm; School of Pharmacy, University of California, San Francisco (AS).

Supplementary table 1: Patterns NSAIDs use in the year prior to cancer diagnosis, further adjustment by lymph-node status. The entries for each period in each table show the number of drug users in the period, the odds ratio (OR) and 95% confidence interval. The OR compares the drug use during each period with the 10-12 month period as reference. The analysis is adjusted for age, sex, education level and lymph-node status.

Breast			
Total	Controls	M0	M1
	95779	13551	394
1-3 mo	6906	1082	86
	0.97(0.94,1.00)	1.03(0.94,1.12)	3.56(2.27,5.59)
4-6 mo	7019	1100	35
	0.98(0.95,1.02)	1.05(0.96,1.14)	1.23(0.73,2.07)
7-9 mo	7184	1057	33
	1.01(0.98,1.05)	1.00(0.92,1.09)	1.15(0.68,1.95)
10-12 mo	7117	1056	29
	1(reference)	1 (reference)	1 (reference)

Prostate			
Total	Controls	M0	M1
	102461	4726	1775
1-3 mo	6029	450	351
	0.96(0.92,0.99)	1.48(1.27,1.72)	3.90(3.10,4.90)
4-6 mo	6023	360	154
	0.96(0.92,0.99)	1.16(0.99,1.36)	1.48(1.15,1.92)
7-9 mo	6009	315	122
	0.95(0.92,0.99)	1.00(0.85,1.18)	1.15(0.89,1.51)
10-12 mo	6284	314	107
	1(reference)	1 (reference)	1 (reference)

Lung			
Total	Controls	M0	M1
	198240	2952	2556
1-3 mo	12935	355	530
	0.96(0.94,0.99)	1.41(1.19,1.67)	2.90(2.44,3.44)
4-6 mo	13042	281	261
	0.97(0.95,1.00)	1.08(0.91,1.29)	1.28(1.06,1.55)
7-9 mo	13193	258	195
	0.98(0.96,1.01)	0.98(0.82,1.18)	0.90(0.74,1.11)
10-12 mo	13401	262	214
	1(reference)	1 (reference)	1 (reference)

Colorectal			
Total	Controls	M0	M1
	198240	9759	2964
1-3 mo	12935	499	256
	0.96(0.94,0.99)	1.01(0.89,1.14)	1.67(1.36,2.06)
4-6 mo	13042	548	173
	0.97(0.95,1.00)	1.11(0.98,1.26)	1.09(0.88,1.37)
7-9 mo	13193	553	169
	0.98(0.96,1.01)	1.12(0.99,1.27)	1.07(0.85,1.33)
10-12 mo	13401	496	159
	1 (reference)	1 (reference)	1 (reference)

Supplementary table 2: Patterns NSAIDs use in the two years prior to colorectal cancer diagnosis, with further adjustment for ulcer and inflammatory bowel disease. The entries for each period in each table show the number of drug users in the period, the odds ratio (OR) and 95% confidence interval. The OR compares the drug use during each period with the 22-24 month period as reference. The analysis is adjusted for age, sex, education level, and ulcer and inflammatory bowel disease.

**Colorectal cancer**			
**Total**	**Controls**	**M0**	**M1**
	148740	7076	2216
**1-3 mo**	9629	362	194
	0.94(0.91,0.97)	0.83(0.72,0.96)	1.49(1.18,1.87)
**4-6 mo**	9657	392	126
	0.94(0.92,0.97)	0.90(0.78,1.04)	0.93(0.72,1.19)
**7-9 mo**	9786	415	129
	0.96(0.93,0.98)	0.96(0.83,1.10)	0.95(0.74,1.22)
**10-12 mo**	9844	358	118
	0.96(0.93,0.99)	0.82(0.71,0.95)	0.87(0.0.67,1.12)
**13-15 mo**	9963	441	127
	0.97(0.95,1.00)	1.02(0.89,1.17)	0.94(0.73,1.20)
**16-18 mo**	9980	428	130
	0.98(0.95,1.00)	0.99(0.86,1.14)	0.96(0.75,1.23)
**19-21 mo**	10192	428	124
	1.00(0.97,1.03)	0.99(0.86,1.14)	0.91(0.71,1.17)
**22-24 mo**	10206	432	135
	1	1	1

Supplementary table 3: Metformin prescription in the year prior to cancer diagnosis. The entries for each period in each table show the number of drug users in the period, the odds ratio (OR) and 95% confidence interval. The OR compares the drug use during each period with the 10-12 month period as reference. The analysis is adjusted for age, sex and education level.

Breast			
Total	Controls	M0	M1
	95779	13551	394
1-3 mo	2875	355	14
	1.04(0.99,1.10)	1.08(0.93,1.22)	1.18(0.53,2.61)
4-6 mo	2876	345	14
	1.04(0.99,1.10)	1.05(0.90,1.22)	1.19(0.53,2.61)
7-9 mo	2760	341	10
	1.00(0.94,1.05)	1.03(0.89,1.21)	0.82(0.35,1.96)
10-12 mo	2768	330	12
	1(reference)	1(reference)	1(reference)

Prostate			
Total	Controls	M0	M1
	102461	4726	1775
1-3 mo	4860	232	79
	1.07(1.02,1.11)	1.20(0.99,1.48)	1.12(0.81,1.55)
4-6 mo	4844	213	87
	1.06(1.02,1.11)	1.10(0.90,1.35)	1.24(0.90,1.71)
7-9 mo	4661	189	79
	1.02(0.98,1.06)	0.97(0.79,1.19)	1.12(0.81,1.55)
10-12 mo	4571	194	71
	1(reference)	1(reference)	1(reference)

Lung			
Total	Controls	M0	M1
	198240	2952	2556
1-3 mo	7735	154	131
	1.06(1.02,1.09)	1.09(0.86,1.38)	1.20(0.93,1.56)
4-6 mo	7720	142	120
	1.05(1.02,1.09)	1.00(0.79,1.27)	1.10(0.84,1.43)
7-9 mo	7421	138	117
	1.01(0.98,1.05)	0.97(0.76,1.23)	1.07(0.82,1.39)
10-12 mo	7339	142	110
	1 (reference)	1 (reference)	1 (reference)

Colon-rectum			
Total	Controls	M0	M1
	198240	9759	2964
1-3 mo	7735	511	127
	1.06(1.02,1.09)	1.01(0.89,1.14)	0.86(0.68,1.10)
4-6 mo	7720	516	141
	1.05(1.02,1.09)	1.02(0.90,1.16)	0.96(0.76,1.22)
7-9 mo	7421	503	148
	1.01(0.98,1.05)	0.99(0.87,1.13)	1.01(0.80,1.28)
10-12 mo	7339	507	146
	1 (reference)	1 (reference)	1 (reference)

Supplementary table 4: Statin prescription in the year prior to cancer diagnosis. The entries for each period in each table show the number of drug users in the period, the odds ratio (OR) and 95% confidence interval. The OR compares the drug use during each period with the 10-12 month period as reference. The analysis is adjusted for age, sex and education level.

Breast			
**Total**	**Controls**	**M0**	**M1**
	95779	13551	394
**1-3 mo**	11685	1392	32
	1.09(1.06,1.12)	1.07(0.98,1.16)	0.77(0.46,1.27)
**4-6 mo**	11497	1358	42
	1.07(1.04,1.10)	1.04(0.96,1.13)	1.07(0.66,1.71)
**7-9 mo**	11124	1308	35
	1.03(1.00,1.06)	0.99(0.91,1.08)	0.86(0.53,1.39)
**10-12 mo**	10864	1316	40
	1 (reference)	1 (reference)	1 (reference)

Prostate			
**Total**	**Controls**	**M0**	**M1**
	102461	4726	1775
**1-3 mo**	18865	963	323
	1.08(1.06,1.10)	1.17(1.05,1.29)	1.07(0.90,1.27)
**4-6 mo**	18611	900	336
	1.06(1.03,1.09)	1.07(0.97,1.19)	1.13(0.95,1.34)
**7-9 mo**	18151	892	306
	1.03(1.01,1.05)	1.06(0.96,1.18)	1.00(0.84,1.20)
**10-12 mo**	17721	851	305
	1 (reference)	1 (reference)	1 (reference)

Lung			
**Total**	**Controls**	**M0**	**M1**
	198240	2952	2556
**1-3 mo**	30550	616	418
	1.08(1.06,1.10)	1.07(0.94,1.21)	1.04(0.90,1.21)
**4-6 mo**	30108	610	429
	1.06(1.05,1.08)	1.05(0.93,1.20)	1.08(0.93,1.25)
**7-9 mo**	29275	570	423
	1.03(1.01,1.05)	0.97(0.85,1.0)	1.06(0.91,1.23)
**10-12 mo**	28585	585	403
	1 (reference)	1 (reference)	1 (reference)

Colorectal			
**Total**	**Controls**	**M0**	**M1**
	198240	9759	2964
**1-3 mo**	30550	1721	406
	1.08(1.06,1.10)	1.06(0.98,1.14)	0.96(0.82,1.11)
**4-6 mo**	30108	1685	419
	1.06(1.05,1.08)	1.03(0.95,1.08)	0.99(0.86,1.15)
**7-9 mo**	29275	1675	413
	1.03(1.01,1.05)	1.02(0.95,1.10)	0.98(0.84,1.13)
**10-12 mo**	28585	1647	422
	1 (reference)	1 (reference)	1 (reference)

Figure S1

Kaplan-Meir curves of overall survival for breast, prostate, lung and colorectal cancer patients who were non-metastatic at the time of primary diagnosis. Exposure is defined as intake of the drug within 6 months prior to cancer diagnosis.

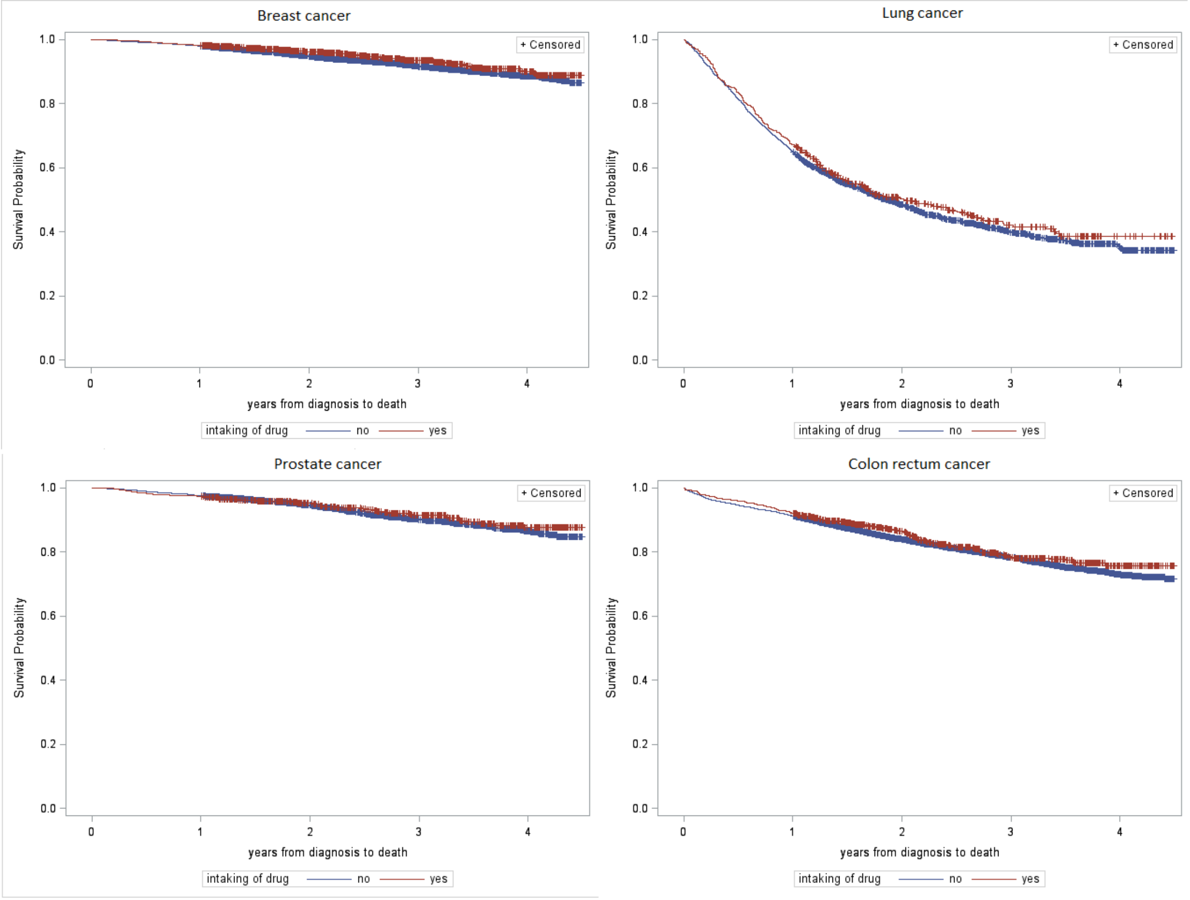

Supplement: Supplementary file 1 — Supplementary Tables [file 41598_2017_133_MOESM1_ESM.pdf]
